# The hops (*Humulus lupulus*) genome contains a mid-sized terpene synthase family that shows wide functional and allelic diversity

**DOI:** 10.1186/s12870-023-04283-y

**Published:** 2023-05-26

**Authors:** Xiuyin Chen, Mindy Y. Wang, Cecilia H. Deng, Ron A. Beatson, Kerry R. Templeton, Ross G. Atkinson, Niels J. Nieuwenhuizen

**Affiliations:** 1 The New Zealand Institute for Plant and Food Research Ltd (PFR), Private Bag 92169, Auckland, 1142 New Zealand; 2 PFR, 55 Old Mill Road, RD 3, Motueka, 7198 New Zealand

**Keywords:** Aroma, **α-**Farnesene, GC–MS, Genome analysis, Linalool, Volatile

## Abstract

**Background:**

Hops (*Humulus lupulus* L.) are a dioecious climbing perennial, with the dried mature “cones” (strobili) of the pistillate/female inflorescences being widely used as both a bittering agent and to enhance the flavour of beer. The glandular trichomes of the bract and bracteole flowering structures of the cones produce an abundance of secondary metabolites, such as terpenoids, bitter acids and prenylated phenolics depending on plant genetics, developmental stage and environment. More knowledge is required on the functional and allelic diversity of terpene synthase (TPS) genes responsible for the biosynthesis of volatile terpenes to assist in flavour-directed hop breeding.

**Results:**

Major volatile terpene compounds were identified using gas chromatography–mass spectrometry (GC–MS) in the ripe cones of twenty-one hop cultivars grown in New Zealand. All cultivars produced the monoterpene β-myrcene and the sesquiterpenes α-humulene and β-caryophyllene, but the quantities varied broadly. Other terpenes were found in large quantities in only a smaller subset of cultivars, e.g. β-farnesene (in seven cultivars) and α-pinene (in four). In four contrasting cultivars (Wakatu™, Wai-iti™, Nelson Sauvin™, and ‘Nugget’), terpene production during cone development was investigated in detail, with concentrations of some of the major terpenes increasing up to 1000-fold during development and reaching maximal levels from 50–60 days after flowering. Utilising the published *H. lupulus* genome, 87 putative full-length and partial terpene synthase genes were identified. Alleles corresponding to seven TPS genes were amplified from ripe cone cDNA from multiple cultivars and subsequently functionally characterised by transient expression *in planta*. Alleles of the previously characterised *HlSTS1* produced humulene/caryophyllene as the major terpenes. *HlRLS* alleles produced (*R*)-(-)-linalool, whilst alleles of two sesquiterpene synthase genes, *HlAFS1* and *HlAFS2* produced α-farnesene. Alleles of *HlMTS1*, *HlMTS2* and *HlTPS1* were inactive in all the hop cultivars studied.

**Conclusions:**

Alleles of four TPS genes were identified and shown to produce key aroma volatiles in ripe hop cones. Multiple expressed but inactive TPS alleles were also identified, suggesting that extensive loss-of-function has occurred during domestication and breeding of hops. Our results can be used to develop hop cultivars with novel/improved terpene profiles using marker-assisted breeding strategies to select for, or against, specific TPS alleles.

**Supplementary Information:**

The online version contains supplementary material available at 10.1186/s12870-023-04283-y.

## Background

Hops (*Humulus lupulus* L.) are vigorous, perennial, dioecious vines that prefer to grow vertically. The *Humulus* genus contains three species, *H. japonicus*, *H. lupulus* and *H. yunnanensis* [1], and belongs to the Cannabaceae family, which also includes hemp and marijuana, *Cannabis indica* and *C. sativa* [1]. The female inflorescences of the hop plant yield the strobili or cones that are used in beer preservation and flavouring as well as in biomedical applications due to the abundance of three major types of secondary metabolites: namely bitter acids (α- and β-), prenylated flavonoids (e.g. xanthohumol and desmethyl-xanthohumol) and terpenoids [2–5]. The α-acids in hops are isomerised during the boil stage in the brewing process and contribute to the bitterness and some of the preservative properties of beer. The prenylated flavonoids such as xanthohumol have been shown to have health benefits such as anti-tumour [6] and anti-inflammatory, anti-bacterial, anti-viral, anti-fungal and anti-plasmodial activities [7]. A number of genes involved in the production of bitter acids, e.g. [8, 9], and prenylated flavonoids, e.g. [10], have been characterised.

Terpenoids in hops also play an important role in beer aroma. β-Myrcene may give beer its ‘green hop’ aroma while the presence or absence of α- and β-farnesene is a distinguishing feature of some hop essential oils. α-Farnesene has a woody, green aroma with a floral nuance, whilst β-farnesene shows woody, citrus and herbal notes. High levels of farnesene in hops generally correlate well with a pleasant, noble-type hop aroma in beer [11]. Linalool is considered a key contributor to ‘hoppy’ flavour and there is a good correlation between fruity, floral flavour and linalool concentration [12]. (*R*)-linalool, has a woody, spicy, and lavender-like character and an odour threshold of less than 1 part per billion (ppb), while (*S*)-linalool has a sweet, floral aroma and a higher aroma threshold of about 7 ppb. Hops can be added at different stages of the brewing process, but in recent years, the trend has increasingly been to deploy them at the later stages to reduce thermal-induced losses of aroma compounds during brewing [13]. The development of new hop cultivars with exceptional aroma sensory characteristics is driven by their use in craft beer production [14].

Terpenoids are derived either from the mevalonate pathway [15, 16], which is active in the cytosol and starts from acetyl CoA, or from the methylerythritol-4-phosphate (MEP) pathway, which is active in the plastids and derived from pyruvate and glyceraldehyde-3-phosphate [17, 18]. These two distinct pathways lead to the formation of the 5-carbon isopentenoid building block isopentenyl diphosphate, which is isomerised to dimethylallyl diphosphate (DMAPP) by isopentenyl diphosphate isomerase [19, 20]. Isoprene, the smallest terpenoid with 5-carbon atoms (C-5/hemiterpene) is produced from DMAPP [21]. DMAPP is also the substrate used by prenyltransferase enzymes for the prenylation steps in bitter acid and prenyl flavonoid biosynthesis. In hops, full-length sequences of the heterodimeric geranyl diphosphate synthase (GPPS) small subunit and large subunit that produce the C-10 GDP substrate for the biosynthesis of monoterpenes have been identified [22], while farnesyl diphosphate synthases (FPPS) have also been identified that produce the C-15 FDP substrate for production of sesquiterpenes [23].

Terpene synthases (TPS) use GDP and FDP as substrates to produce the great diversity of volatile aroma terpene skeletons with around 1400 different mono- and sesquiterpenes reported [24]. TPS genes exist in medium to large gene families derived from eight clades of distinct evolutionary lineages (TPSa-h) [25]. Accompanying this gene family expansion, the TPS enzymes also show great functional plasticity, where minor active site alterations can dramatically affect product outcomes enabling the emergence of new alleles and gene functions [26, 27]. In cannabis, genome-wide analysis of the ‘Purple Kush’ reference genome identified nineteen complete CsTPS gene models, and tandem arrays of isoprenoid and cannabinoid biosynthetic genes [28]. In the hop cultivar ‘Phoenix’, a monoterpene synthase (*HlMTS2*) has been characterised that produces the monoterpene β-myrcene, and a sesquiterpene synthase (*HlSTS1*) that produces a mixture of α-humulene and β-caryophyllene (H:C) in an approximately 3:1 ratio [29]. A germacrene-A synthase gene (*HlSTS2*) with 92% amino acid (AA) identity to *HlSTS1* has also been reported [29].

New Zealand grows many locally bred hop cultivars including Wakatu™ and Wai-iti™, and also important European and American cultivars such as ‘Wye Challenger’ and ‘Cascade’ (https://grainfather.com/new-zealand-hops/). One of the contrasting and unique features of New Zealand-bred hops has been the breeding of triploid cultivars, which are beneficial in producing largely seedless cones. The flavour attributes of European and American cultivars grown in New Zealand can differ considerably from the same cultivars grown elsewhere, due to different environmental and growing conditions. Differences in ‘terroir’ affect the qualitative and quantitative composition of hop volatile oils [30] and economic value can be added to beers brewed with hops associated with specific local or regional identities [31]. ‘Cascade’ hops grown in New Zealand are marketed as Taiheke® (www.nzhops.co.nz) and ‘NZ Cascade’ to reflect this difference in flavour attributes associated with the change to New Zealand terroir.

In this study we investigate the terpene aroma compounds produced during cone development by a range of hop cultivars grown under New Zealand conditions. Our aim was to identify and characterise novel TPS genes that could account for the differences in terpene profiles in novel hop cultivars such as Wakatu and Wai-iti through an integrated analysis of metabolomics profiles with recently published genomic and transcriptomic resources [32, 33].

## Results

### Hop cultivars used in this study

Of the 265 different hop cultivars around the world [34], we selected a representative sample of twenty-one cultivars, which were grown as part of the Core Hops Germplasm Collection at The New Zealand Institute for Plant and Food Research Limited (PFR) Motueka Research Station for analysis. Motueka is situated in the main region for commercial hop production in New Zealand (41.1081° S, 173.0112° E). Six of the cultivars analysed were bred in New Zealand, while the other fifteen cultivars originated from countries including the USA, Germany, Australia, UK and Slovenia (Table 1). The six cultivars from New Zealand are all triploid, which promotes seedlessness, whilst fourteen of the fifteen cultivars originating overseas are diploid. Previous studies have shown that the twenty-one selected cultivars display considerable differences in the chemical composition of steam-distilled oil extracts [34] and in α- and β-acid composition. For example, Moutere™ has the highest α-acid (17–20%) and β-acid (8–10%) content, whilst ‘Hersbrucker Spät’ has amongst the lowest (α-acid 2–5%; β-acid 4–6%) (Table 1).

**Table 1:** Published characteristics of the twenty-one hop cultivars used in this study. Ploidy levels were determined by flow-cytometry. D = diploid; T = triploid. Total oil content was measured by high performance liquid chromatography (HPLC) from water distilled steam oil extracts. nd = no data available

Cultivar	Abbreviation	Origin	Ploidy level	α-acid (%)	β-acid (%)	Cohumulone (%)	Total oil (mL 100 g−1)	Myrcene (% in oil)	Humulene (% in oil)	Caryophyllene (% in oil)	Farnesene^a^ (% in oil)	Reference^b^
‘Aquila’	Aqu	USA	D	6.7–8.9	4.1–4.9	46	1.45	62	2	5	2.2	1
‘Cascade’/Taiheke®	Cas	USA	D	4.5–8.9	3.6–7.5	33–40	0.8–1.5	45–60	8–16	4–6	4–8	1
‘Hallertauer Magnum’	Ham	Germany	D	12–14	4.5–5.5	24–25	19–2.3	30–35	34–40	8–12	0–1	1
‘Hersbrucker Spät’	Hes	Germany	D	2–5	4–6	19–25	0.5–1.3	10–25	15–35	7–15	0–1	1
‘Liberty’	Lib	USA	T	3–6	3.5	24–28	1.3	46	31	9–12	< 1	1
Motueka™	Mot	NZ	T	6.5–8.5	5–5.5	29	0.8	47.7	3.6	2	12.2	1
Moutere™	Mou	NZ	T	17.5–19.5	8–10	26	1.7	22.2	15.2	5.8	0.3	2
Nelson Sauvin™	Nes	NZ	T	12–13	6–8	24	1.1	22.2	36.4	10.7	0.4	1
‘Nugget’	Nug	USA	D	9.5–14	4.2–5.8	22–30	1.5–3	48–59	12–22	7–10	0–1	1
‘Perle’	Per	Germany	D	8–9	8	28	0.6–1.2	44	29	10.2	0.2	1
‘Pride of Ringwood’	Por	Australia	D	7–11	4–8	33–39	1–2	25–53	2–8	5–10	< 1	1
Riwaka™	Riw	NZ	T	4.5–6.5	4–5	29–36	0.8	68	9	4	1	1
‘Talisman’	Tal	USA	D	5.7–6.7	2.8–3.6	53	0.72	68	4	5.9	0.2	1
‘Target’	Tar	UK	D	8–12.5	5–5.5	29–35	1.6–2.6	17–22	8–10	0–1	0–1	1
‘Tasmanian Gold’	Tag	Australia	D	nd	nd	nd	nd	nd	nd	nd	nd	nd
‘Tettnanger A’	Tet	Germany	D	3–5.8	2.8–5.3	24	0.36–1.07	40.6	20.4	6.2	11.3	1
‘USDA21055’	Usd	USA	D	14.7	5.7	42–45	2	nd	nd	nd	nd	3
Wai-iti™	Wai	NZ	T	2.5–3.5	4.5–5.5	22–24	1.6	30	28	9	13	1
Wakatu™	Wak	NZ	T	6.5–8.5	8.5	28–30	1	36	17	8	6.7	1
‘Wye Challenger’	Wch	UK	D	6.5–9	3.2–4.5	20–25	1–1.7	30–42	25–32	8–10	1–3	2
‘Yugo 27P04’	Yug	Slovenia	D	nd	nd	nd	nd	nd	nd	nd	nd	nd

^a^The isomeric composition of farnesene is not stated in the references used. It is reported as “percentage farnesene in oil”

^b^ 1: Healey J: The Hops List. 265 beer varieties from around the world., 1 edn. self-published; 2016; 2: http://www.nzhops.co.nz; 3: Hounold A, Likens ST, Nickerson GB, Horner CE, Zimmermann CE: Registration of USDA 21055 Hop Germplasm 1 (Reg. No. GP 5). *Crop Science* 1978, 18(5):919

### Survey of volatiles produced by twenty-one hop cultivars grown under New Zealand conditions

**Fig. 1:**
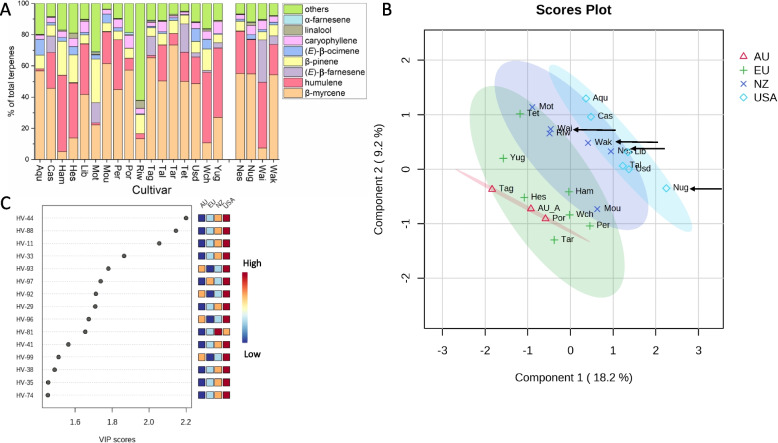
Volatiles produced by twenty-one hop cultivars grown in New Zealand. **A** Total volatile terpenes (%) were collected by SPME and analysed by GC–MS. Cultivar abbreviations are given in Table 1. The four cultivars studied in detail during cone development are separated on the right. **B** Partial Least Squares-Discriminant Analysis (PLS-DA) of the volatiles measured by SPME. Complete volatile measurement data and a correlation matrix are given in Table S1A and B. Cultivar origin: Au – Australia (red); NZ—New Zealand (dark blue); USA—United States of America (light blue); Eu – Europe (green). *Au_A: Average of Tag and Por. 95% confidence regions are shown by the coloured spheres. The four cultivars studied in detail during cone development are arrowed. **C** Compounds driving the discrimination in the PLS-DA. Component 1 VIP scores for the top 15 compounds: HV-44: trans-β-ocimene, HV-88: (*Z*,*E*)-α-farnesene, HV-11:, α-pinene, HV-33: limonene, HV-93: γ-cadinene, HV-97: 2-tridecanone, HV-92: delta-cadinene, HV-29: α-terpinene, HV-96: cadinene isomer, HV-81: 4-decenoic acid, ethyl ester, (*E*)-, HV-41: ethyl 5-methylhexanoate, HV-99: geraniol, HV-38: cis-β-ocimene, HV-35: β-phellandrene, HV-74: linalool

Ripe cone samples of the twenty-one selected hop cultivars were collected in the same harvest year. Over 100 volatile compounds were detected in the representative pooled sample from each cultivar using headspace solid-phase micro-extraction (SPME) followed by gas chromatography–mass spectrometry (GC–MS). The main compounds were terpenes, esters and sulfur volatiles (Table S1A), with terpenes being the most abundant (> 70% of the volatile organic compounds, VOCs) in most of the hop cultivars surveyed. Thirty-six terpene compounds were detected across the panel, with the percentage abundance of individual terpene compounds varying greatly between cultivars (Table S1A, Fig. 1A). β-Myrcene and α-humulene were the dominant terpene volatiles in most cultivars. ‘Liberty’, ‘Nugget’ and Riwaka™ produced the most linalool (> 6 µg∙g^−1^ FW). About two thirds of the cultivars produced a little β-farnesene, while seven cultivars produced > 20 µg∙g^−1^ FW. ‘Pride of Ringwood’ was the highest producer of α-farnesene isomers (> 6 µg∙g^−1^ FW). Ratios of α-humulene: β-caryophyllene (H:C) varied widely from 0.5 in ‘Aquila’ to 12 in ‘Hallertauer Magnum’ (Table S1A). The α-humulene and β-caryophyllene ratio for ‘Nugget’ ripe cones measured by SPME (H:C = 3.75) approximates that previously described in hexane extracts (H:C = 3) [29].

Analysis of total volatile profiles (Table S1A**)** by Partial Least Squares-Discriminant Analysis (PLS-DA) indicated 28.9% of the variation could be explained in the first two dimensions (Fig. 1B). The USA-bred cultivars were separated from the European + Australian cultivars, while the New Zealand cultivars overlapped with the other three regions of origin (Fig. 1B). The compounds driving the separations were mainly terpenes and esters but also included ketones (Fig. 1C) in the top 15. Terpenes such as linalool, β-ocimene and α-farnesene were amongst the most important driving the discrimination. Among the ketones, 2-tridecanone (HV-97) was in the top 15, as were the esters ethyl (*E*)-4-decenoate (HV-81), and ethyl 5-methylhexanoate (HV-41).

### Terpene volatile production during cone development in four hop cultivars

**Fig. 2:**
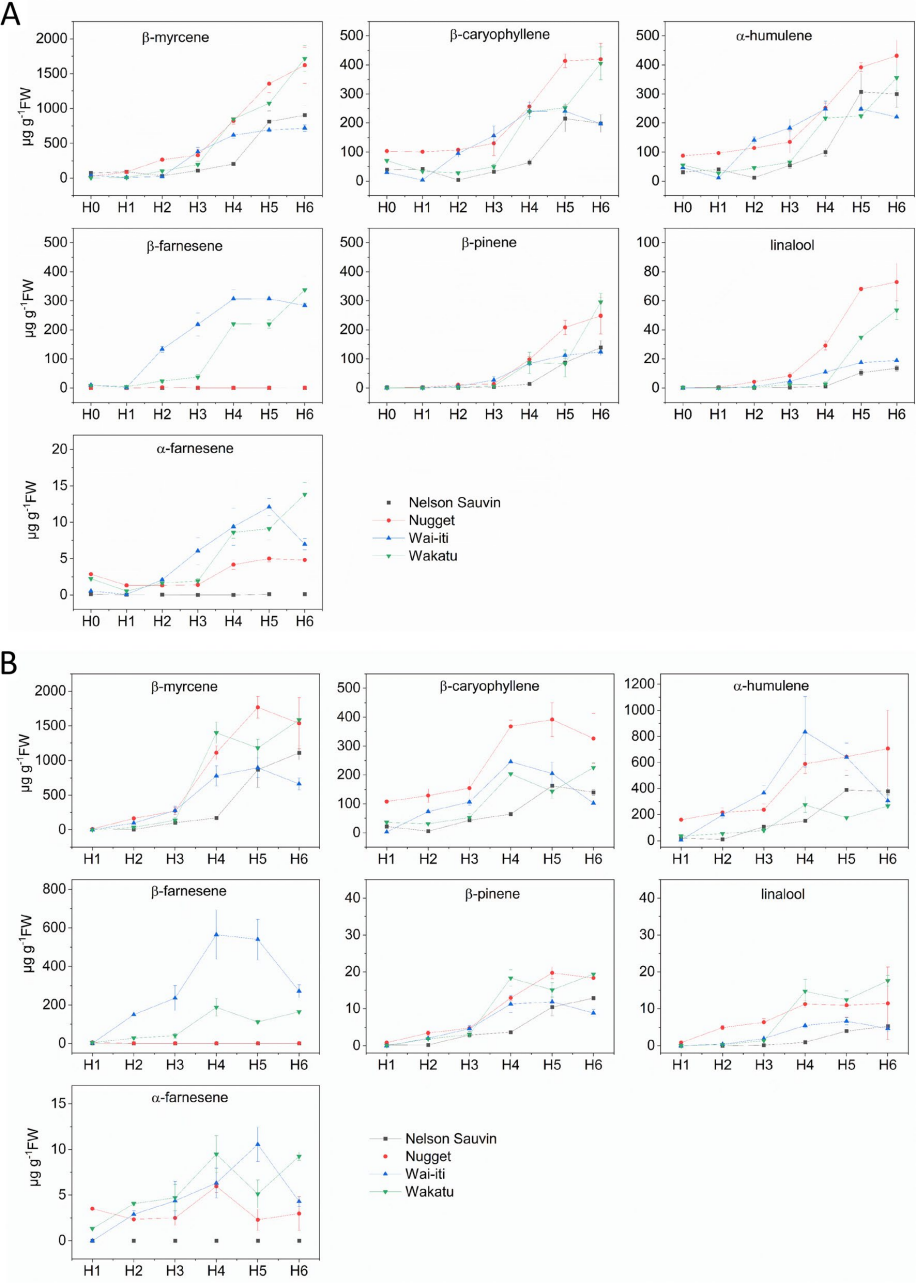
Seven key volatile terpenes produced in four hops cultivars during cone development. **A** Volatiles collected by SPME and analysed by GC–MS. **B** Volatiles collected by solvent extraction and analysed by GC–MS. Data are shown as mean ± SE; *n* = 3. Samples at H0 were collected 10 days after anthesis (DAA). H1-H7 were collected at 10 d intervals from 20 to 70 DAA. Data are displayed in µg∙g^−1^ FW

To gain a better understanding of the temporal changes in terpene production during cone development, hop cones from four cultivars (Nelson Sauvin, ‘Nugget’, Wai-iti, Wakatu, were sampled at 10-day intervals (seven sampling points H0-H6; Fig. 2) from 10 days after flowering (DAF) through to commercial harvest stage. Samples were measured by two complementary analytical methods, SPME and solvent extraction. In all four cultivars, monoterpene and sesquiterpene concentrations typically increased slowly from commencement of flowering to H2 (30 DAF), and rapidly through H3-H5 (40 to 60 DAF) (Fig. 2). Overall, the concentrations of the most abundant volatiles, and the patterns detected by SPME and solvent extraction were similar (Fig. 2A and B), with some minor exceptions.β-Myrcene and β-pinene accumulation patterns were very similar during cone development across the four cultivars (Fig. 2). Interestingly, the α-humulene: β-caryophyllene ratio (H:C) changed considerably across development, especially in Nelson Sauvin and Wai-iti and the ratio was much lower for SPME (H:C ratio of 1.25 averaged across all time points and all four cultivars) versus solvent extraction (H:C average 2.01) (Fig. 2A vs. B). Differences were also observed among cultivars in the content of terpenes through development (Fig. 2A). Wai-iti started to produce a high amount of β-farnesene as early as H2 and reached 307 µg∙g^−1^ at harvest H4 and H5 (SPME). The level of β-farnesene in Wai-iti at H4 (solvent) was 565 µg∙g^−1^ FW, which is threefold higher than H4 Wakatu (Fig. 2B), while with SPME the levels were only 1.4-fold higher at H4 and H5. In contrast, Nelson Sauvin and ‘Nugget’ produced very little β-farnesene at H4 and H5. The levels of β-pinene increased 18-, 11-, 13- and fourfold from H1 to H4 for Wakatu, Wai-iti, ‘Nugget’ and Nelson Sauvin, respectively. β-Myrcene reached 1400 µg∙g^−1^ FW at H4 for Wakatu compared with only 1 µg∙g^−1^ FW at H1 (Fig. 2A). For the solvent extraction, the content of ß-myrcene increased 778-, 1111- and 174-fold from H1 to H4 for Wai-iti, ‘Nugget’ and Nelson Sauvin, respectively (Fig. 2B).

### Terpene synthase identification in the *H. lupulus* ‘Cascade’ genome

Four TPS genes have previously been characterised from hop trichomes [29]: two sesquiterpene synthases *HlSTS1* (α-humulene/ β-caryophyllene synthase; H:C ratio 2.8:1) and *HlSTS2* (germacrene A synthase), one monoterpene synthase *HlMTS2* (β-myrcene synthase), and one monoterpene-like gene of unknown function, *HlMTS1* (Table S2A); all derived from the cultivar ‘Phoenix’. These four genes were all expressed during cone development and in hop trichomes [29]. Enzymes for synthesis of other major and minor sesquiterpene and monoterpene compounds, such as α- and β-farnesene as well as linalool, α-pinene, β-pinene, α-terpinene, limonene, β-phellandrene and β-ocimene have not been reported.

The release of a high-quality *H. lupulus* ‘Cascade’ genome sequence [32, 33] provides new opportunities to identify and characterise additional terpene synthases important to hop flavour and aroma. A homology-based gene mining approach was undertaken to identify putative TPS genes that could account for the production of terpene compounds in hop cones. Using *C. sativa* terpene synthase (TPS) gene amino acid sequences as queries [35] and searching against the ‘Cascade’ gene models (http://hopbase.cgrb.oregonstate.edu/) [32, 33], eighty-seven gene models were found with sizes ranging from 471 to 4227 bp (Table S3). Thirty-six putative full-length TPS were identified, of which eighteen likely contained both a complete N- (ATG start) and C-terminal (stop codon) region (Table S3). None of these gene models showed 100% identity to any of the full-length published hop TPS sequences. However, several incomplete gene models displayed high sequence similarity, with homologies ranging from 80 to 100% identity across the aligned amino acid sequence regions (Table S3, notes/known hop TPS). Eight (22%) of the 36 putative full-length monoterpene synthase gene models contained predicted chloroplast targeting peptides (https://services.healthtech.dtu.dk/service.php?ChloroP-1.1) and this feature was represented in 12% of the 87 gene models (Table S3). Known conserved TPS motifs, including RR(X8)W (21%) and DDXXD (58%) were also identified in some gene models.

### Cloning of terpene synthases from ripe hop cones

PCR primers were designed to amplify novel alleles of three previously published HlTPS genes from the cultivar ‘Phoenix’: *HlMTS1-Phx* (unknown function), *HlMTS2-Phx* (β-myrcene synthase) and *HlSTS1-Phx* (α-humulene/ß-caryophyllene synthase) using ripe cone cDNA from ‘Cascade’/Taiheke (Cas), Wai-iti (Wai) and/or Wakatu (Wak). Several alleles corresponding to *HlMTS1-Phx* (EU760348) were amplified and sequenced. None of the alleles were 100% identical in length compared with the published sequence. For example, *HlMTS1*-*Wai-a* had a deletion of approximately 128 AAs starting after AA 53 (Table S2B). For *HlMTS2* (EU760349; myrcene synthase), *HlMTS2-Wai-a* contained nine nucleotide (nt) changes, resulting in seven AA substitutions compared with *HlMTS2-Phx*. *HlMTS2-Wai-b* had two nt changes compared with *HlMTS2-Phx*, which resulted in one AA substitution (F508L) and both alleles contained an uninterrupted open reading frame (ORF). When cloning *HlSTS1*, *HlSTS1-Wai-a* showed 2 nt/2 AA substitutions compared with *HlSTS1-Phx* (EU760350) (Table S2B).

Primers were also designed to amplify four additional TPS gene models: *HlTPS1* (000576F.g12.t3), *HlTPS2* (001370.g12.t1,), *HlTPS3* (001920F.g4.t1) and *HlTPS4* (004063F.g22.t1) with the aim to identify novel TPS genes with unknown activities that are expressed in ripe cone tissue. Using *HlTPS1-4* primers, bands of approximately 1800 bp were amplified and cloned into the plant transient expression vector pHEX2 [36] – a Gateway cloning (Invitrogen) compatible plant expression vector driven by a 35S CaMV promoter and harbouring an octopine synthase terminator.

For *HlTPS1*, two bands were amplified from ripe cone cDNA of approximately 1700 and 1800 bp. Wakatu cDNA yielded two different putatively full-length alleles, *HlTPS1-Wak-a* and* -b*, which encoded 607 AA ORFs and contained predicted (TargetP) chloroplast targeting signals characteristic of monoterpene synthases. Wai-iti yielded three alleles that were likely inactive (Table S2C). *HlTPS1-Wai-a* showed a 37 AA deletion spanning the conserved DDXXD TPS domain. *HlTPS1-Wai-b/c* potentially encoded a 607 AA ORF, but contained a 1 bp internal frameshift, which resulted in a premature stop.

For *HlTPS2*, a single allele was obtained from Wai-iti (*HlTPS2*-*Wai-a*) encoding a protein of 589 AA that was identical to the 001370F.g12.t1 ‘Cascade’ gene model. The protein sequence contained a predicted (TargetP) chloroplast targeting peptide characteristic of monoterpene synthases and both an RRX8W and DDXXD conserved motif in the N- and C-terminal domain, respectively. An identical allele was cloned from Wakatu (*HlTPS2-Wak-a*), while a second allele *HlTPS2-Wak-b*, showed a single AA substitution (L121F) (Table S2C).

*HlTPS3-Cas-a*, was amplified from ‘Cascade’/Taiheke and encodes a protein of 585 AA that contained both RRX8W and DDXXD motifs in N- and C-terminal domain, respectively. This gene lacked a predicted plastid-targeting signal, suggesting it may act as a sesquiterpene synthase. Nearly identical alleles, designated *HlTPS3*-*Wak-a* and *HlTPS3*-*Wai-a*, were amplified and cloned from Wakatu and Wai-iti (Table S2C). The alleles shared > 99% DNA identity (see alignment Figure S1A/B).

A fourth gene designated *HlTPS4*-*Cas-a*, encoding a 587 AA protein, was amplified from ‘Cascade’/Taiheke cDNA (Table S2C). *HlTPS4*-*Cas-a* shows 94% AA identity with the three *HlTPS3* alleles, and 100% nt identity to the 004063F.g22.t1 gene model. All genes and alleles cloned are summarised in Table S2.

### TPS expression levels in the ripe cones of twenty-one hop cultivars

**Fig. 3:**
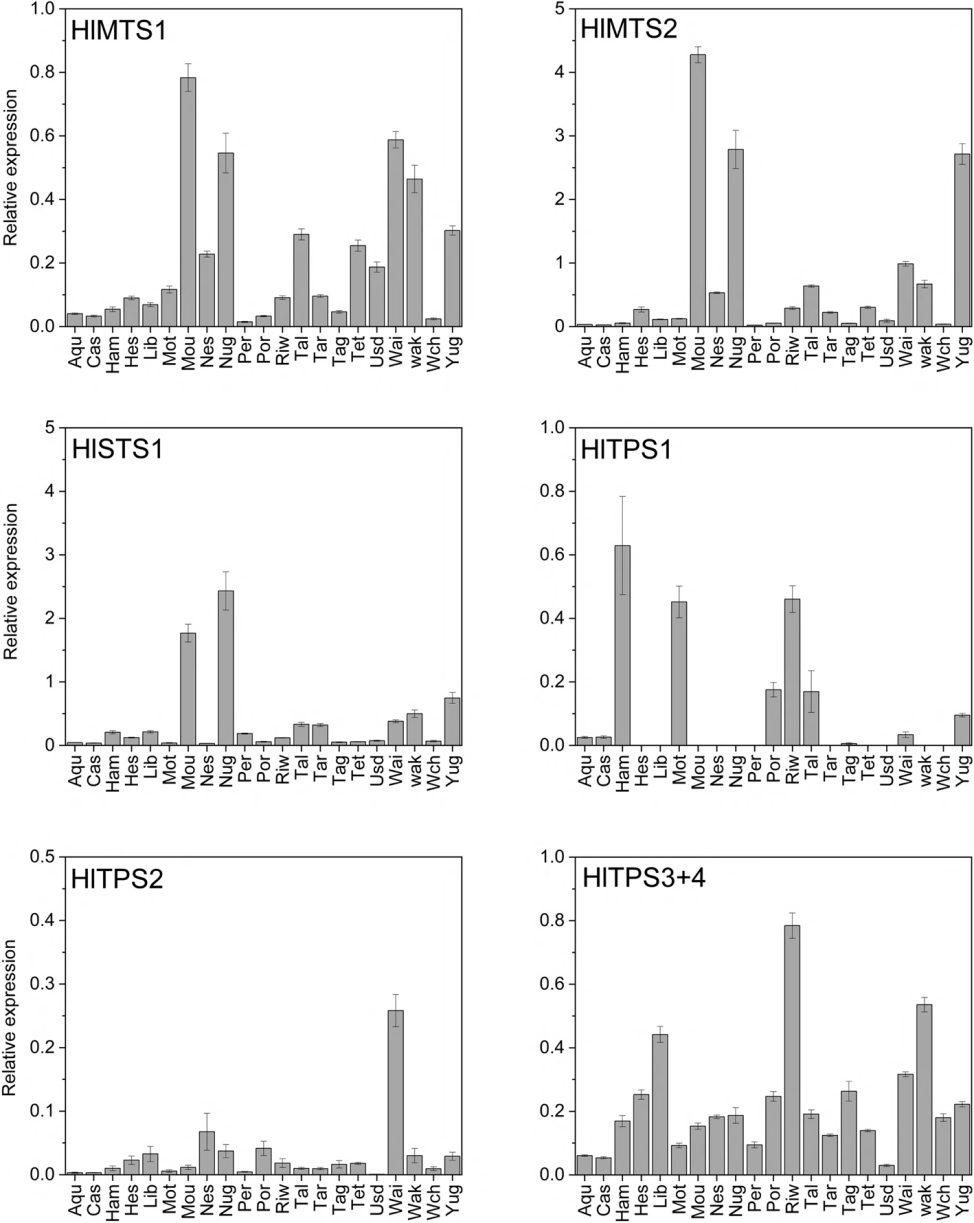
qRT-PCR analysis of seven cloned TPS genes in ripe cones of twenty-one hops cultivars. Cultivar abbreviations are given in Table 1. Gene expression data are mean ± SE, *n* = 3, and are given relative to reference gene *DRH1*. Gene-specific primers are listed in Table S5

Expression of the seven cloned TPS genes was assessed in ripe cones at harvest time (Fig. 3) using quantitative real time PCR (qRT-PCR). Gene-specific primers were designed to the published TPS genes *HlMTS1*, *HlMTS2* and *HlSTS1*, as well as to the novel TPS genes *HlTPS1* and *HlTPS2.* A set of primers was designed to amplify *HlTPS3* + *4* combined, as high sequence identity precluded the design of gene-specific primers for *HlTPS3* and *-4* separately.

*HlMTS1* (unknown function) was moderately expressed in Moutere, ‘Nugget’, Wai-iti and Wakatu. *HlMTS2* (β-myrcene) and *HlSTS1* (α-humulene/ β-caryophyllene synthase) were highly expressed in ripe Moutere, ‘Nugget’ and ‘Yugo’ (Fig. 3). For the newly identified TPS genes, *HlTPS1* showed moderate expression in ‘Hallertauer Magnum’, Motueka™ and Riwaka. *HlTPS2* was expressed moderately in Wai-iti. For *HlTPS3* + *4*, expression levels were highest in Riwaka, Wakatu and ‘Liberty’ and lowest in ‘USDA21055’, ‘Aquila’ and ‘Cascade’.

### Gene expression during cone development in four hop cultivars

**Fig. 4:**
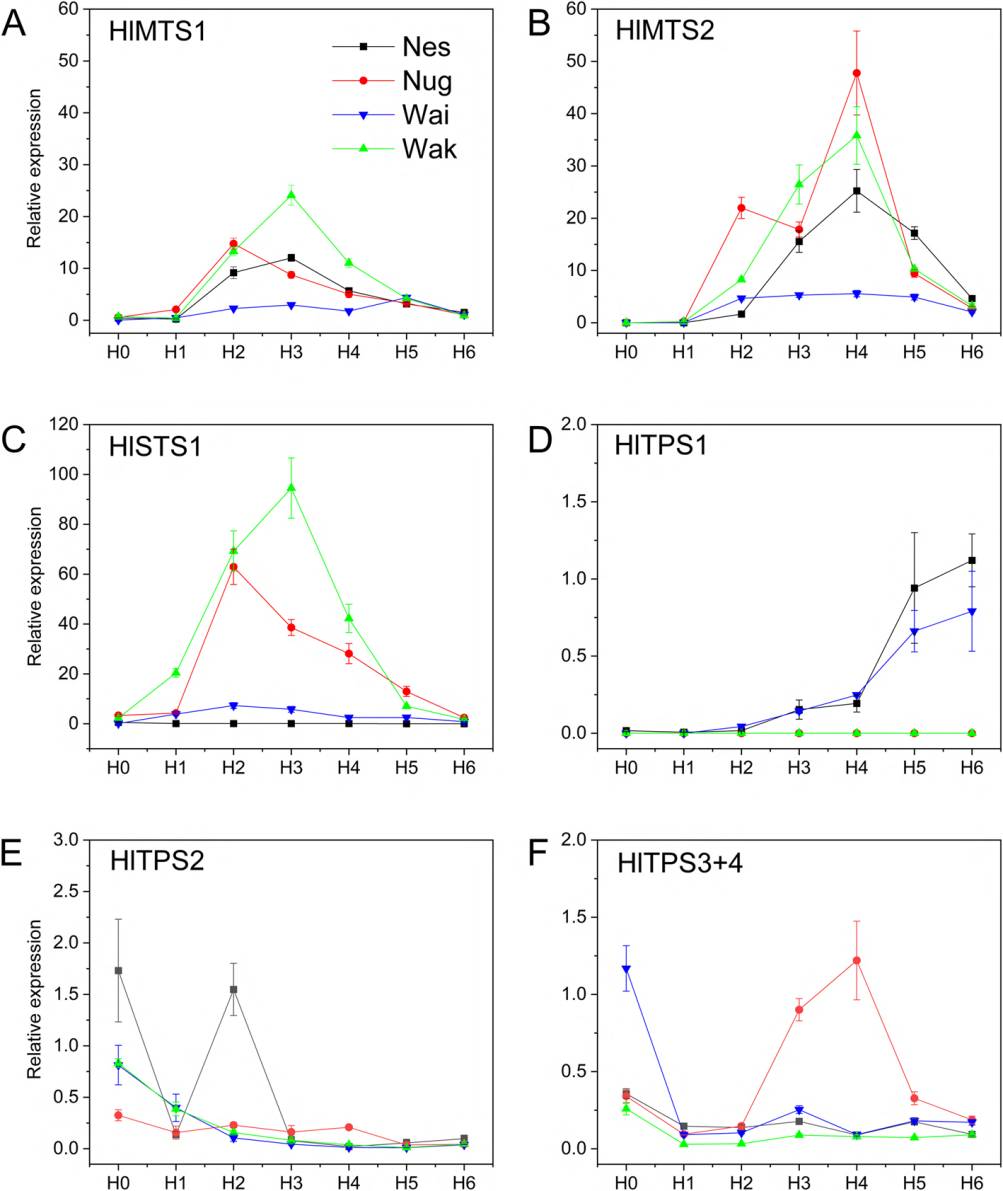
qRT-PCR analysis of seven cloned TPS genes in four hops cultivars during cone development. Cultivar abbreviations are given in Table 1. Gene expression data are mean ± SE, *n* = 3 and are given relative to reference gene DRH1 [37, 38]. Gene-specific primers are listed in Table S5. Samples at H0 were collected 10 days after flowering (DAF). H1-H6 were collected at around 10 d intervals from 20–70 DAF

qRT-PCR was used to assess the expression of the seven cloned TPS genes (Fig. 4) during cone development in four cultivars, Nelson Sauvin, ‘Nugget’, Wakatu and Wai-iti to determine their potential for contributing to volatile production. The samples used for analysis (starting from young cones (H0) up to harvest (H6)) corresponded to those used for volatile analysis by GC–MS in Fig. 2. Expression of the three published terpene synthases and the four newly identified terpene synthases increased as the cones developed, and showed different patterns of expression depending on the gene and cultivar (Fig. 4). Generally, expression was low at the H0 and H1 stages and increased in the later stages.

*HlMTS1* expression reached a peak at H2/H3 for Wakatu, Nelson Sauvin and ‘Nugget’, but at H5 for Wai-iti and showed much lower peak levels in Wai-iti than the other three cultivars. *HlMTS2* (β-myrcene synthase) transcript level peaked for ‘Nugget’, Wakatu and Nelson Sauvin at H4 and also showed a much lower level of expression in Wai-iti. Sesquiterpene synthase gene expression showed quite different patterns (Fig. 4). *HlSTS1* (α-humulene and β-caryophyllene synthase) reached very high expression levels at H3 in Wakatu and H2 in ‘Nugget’ but was lower in Wai-iti and virtually absent in Nelson Sauvin although considerable amounts of α-humulene and β-caryophyllene were produced (Fig. 2) by this cultivar.

*HlTPS1* expression level increased as the cone maturity increased in Nelson Sauvin and Wai-iti, but was virtually absent in Wakatu and ‘Nugget’ (Fig. 4D). Transcript levels of *HlTPS2* were highest at H0 for all four cultivars (Fig. 4E) but a second peak of expression was observed at H2 for Nelson Sauvin. Expression decreased toward maturity (after H3) in all four cultivars and was lowest at H5 and H6. *HlTPS3* + *4* expression declined from H0 to H1, then increased in ‘Nugget’ at H3 and H4, and declined thereafter. Expression in Wai-iti, Wakatu and Nelson Sauvin was highest at H0 and lower during the later stages (Fig. 4E) with Wai-iti showing the highest expression at H0.

### Functional characterisation of hop terpene synthases

Transient *Agrobacterium tumefaciens*-mediated *in planta* TPS expression [36] was used to investigate the terpenes produced by the previously studied genes *HlSTS1* and *HlMTS2* as positive controls and the novel genes *HlTPS1-4*. Infiltrations were performed in combination with either 1-deoxy-D-xylulose-5-phosphate synthase (DXS) (MEP pathway – monoterpenes) or 3-hydroxy-3-methylglutaryl-coenzyme A reductase (HMGR) (mevalonate pathway – sesquiterpenes) with the aim to increase substrate production (GDP and FDP, respectively). Infiltrated leaves were detached from *Nicotiana benthamiana* plants 5 days after *Agrobacterium* infiltration, volatiles collected by SPME, and analysed by GC–MS. As a negative control, volatiles were measured from leaves infiltrated with a binary vector expressing the GUS reporter gene or the substrate pathway genes DXS or HMGR only (Table S4).

**Fig. 5:**
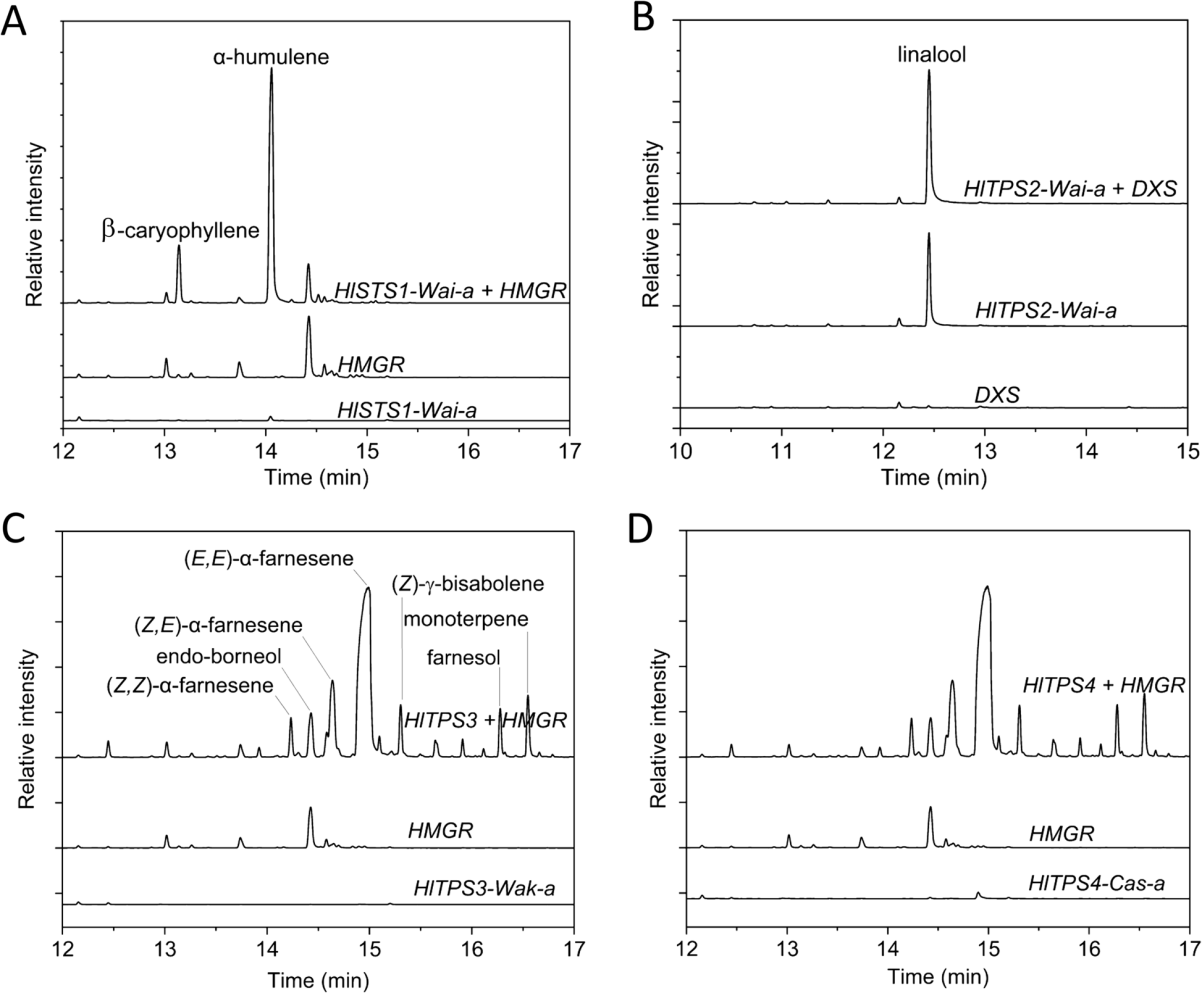
Functional analysis of TPS genes from hops by transient expression in *Nicotiana benthamiana* leaves. DXS/HMGR were co-infiltrated to boost substrate supply for terpene biosynthesis. Leaves were infiltrated with TPS + substrate gene, or a 1:1 mixture of TPS and GUS/substrate gene and GUS for control infiltrations, respectively. Volatiles were collected by SPME and analysed by GC–MS. A representative trace is shown for one allele from each of the four active TPS genes. Full data are presented in Table S4. **A** Terpenes produced by *HlSTS1-Wai-a* in combination with *HMGR* (upper trace) compared to controls *HMGR* only (middle trace) and *HlSTS1-Wai-a* only (lower trace). **B **Terpenes produced by *HlTPS2-Wai-a* + DXS (upper trace) compared to controls *HlTPS2-Wai-a* only (middle trace) and DXS only (lower trace). **C** Terpenes produced by *HlTPS3-Wak-a* combined with *HMGR* (upper trace) compared to controls *HMGR* only (middle trace) and *HlTPS3-Wak-a* only (lower trace). **D** Terpenes produced by *HlTPS4-Cas-a* combined with *HMGR* (upper trace) compared to controls *HMGR* only (middle trace) and *HlTPS4-Cas-a* only (lower trace)

*HlSTS1-Wai-a* produced α-humulene and β-caryophyllene as the major sesquiterpenes (Fig. 5A; Table S4). The H:C ratio for this allele from ‘Wai-iti’ quantified using SPME GC–MS analysis was somewhat higher (4.6), compared with that reported for *HlSTS1*-*Phx* (2.8) using solvent extraction [29] and may partially reflect the different sampling methods. Both alleles of *HlMTS2-Wai-a* and *-b* appeared inactive and did not produce any terpenes at higher levels than the GUS control, even though this gene (*HlMTS2-Phx*) was previously characterised in vitro and shown to produce β-myrcene [29]. These results were negative, regardless of the presence or absence of DXS (Table S4). *TPS1*-*HlTPS1*-*Wak-a* and *-b* alleles were also inactive when expressed transiently *in planta* (Table S4).

Plants infiltrated with the *HlTPS2* binary constructs (*HlTPS2*-*Wai-a*/*Cas-a*) produced linalool as the major monoterpene (Table S4), but this was not apparent for *HlTPS2*-*Wak-b*. The results for *HlTPS2*-*Wai-a* are shown in Fig. 5B. Smaller amounts of β-myrcene, β-ocimene, limonene and linalool oxides were also produced and co-infiltration with DXS did not further increase linalool production (Table S4). Enantiomeric analysis of linalool produced in the *N. benthamiana* leaves infiltrated with *HlTPS2*-*Wai-a* showed that only the (*R*)-(-)-linalool isomer was produced (Table 2). This corresponds well with the enantiomeric composition of linalool in ripe hop cones, which also produce predominantly (> 90%) the (*R*)-(-)-linalool enantiomer (Table 2). *HlTPS2* was therefore designated *HlRLS1* for *H. lupulus R*-linalool synthase 1.

**Table 2:** Linalool enantiomer identification in ripe cones of eight selected hop cultivars

Cultivar or gene	(*R*)-(-)-linalool (%)	(*S*)-( +)-linalool (%)
‘Harley's Fulbright’	88.3	11.7
‘Smooth Cone’	91.6	8.4
‘Calicross’	87.2	12.8
Wakatu™	92.0	8.0
‘Dr Rudi’	93.4	6.6
Green Bullet™	92.0	8.0
‘First Choice’	91.5	8.5
‘Cascade’/Taiheke®	91.0	9.0
*HlRLS1*^*a*^	100	0

^a^ = linalool enantiomers produced by *HlRLS1* after transient overexpression in *Nicotiana benthamiana*

Leaves infiltrated with *HlTPS3* alleles *HlTPS3*-*Wak-a/Wai-a/Cas-a* as well as *HlTPS4* from ‘Cascade’/Taiheke (*HlTPS4*-*Cas-a*) all produced large amounts of (*E*, *E*)-α-farnesene as the major product (Fig. 5C and D; Table S4) with small amounts of other terpenes. This was most apparent when co-infiltrated with HMGR, which strongly increased (*E*, *E*)-α-farnesene production for both genes. Sesquiterpene production was very low with the TPS gene only, and HMGR or GUS controls. *HlTPS3* and *HlTPS4* were designated *HlAFS1* and *HlAFS2,* respectively, for *H. lupulus* α-farnesene synthase 1 and 2.

## Discussion

Hop cones produce large amounts of mono- and sesquiterpenes that contribute to the aroma properties relevant for beer brewing [29, 34, 41]. Here we show that each of the twenty-one hop cultivars grown in New Zealand that we analysed produces a unique blend of terpenes at harvest (Table 1). The qualitative and quantitative differences in terpene composition between hop cultivars depends in part on the genetic makeup of each cultivar but is also likely to be influenced by environmental factors and cultivation practices (weather, climate, soil, fertilisers, irrigation, plant age etc.) [42]. Terpenes such as β-myrcene and α-humulene/ β-caryophyllene were found in all hop cultivars at varying levels and ratios (Table S1A). In contrast, α-and β-farnesene and linalool were found only in a subset of hop cultivars (Table S1A) [43]. Chiral analysis showed that most of the linalool (> 90%) found in hops was the R-isomer as has been reported previously [44]. In addition to being important in the brewing process, these terpene compounds and other terpenes found in hop cones are likely to be involved in ecological interactions such as in defence against pests and pathogens, plant-plant interactions or may be of benefit to the spread/survival of seeds[45–47].

Terpene synthases are the metabolic gatekeepers in the production of C10 and C15 terpenoids. Some terpene synthases are specialised to produce strictly one terpene, while others are multi-product enzymes [48]. In the *C. sativa* genome at least 30 TPS genes have been identified and functionally characterised with variations of expression and function contributing to the different terpene profiles observed in different cannabis cultivars [48]. In hops, this area of research is still awaiting full exploration. The publication of a high quality hops genome, combined with detailed characterisation of the TPS family members in the *H. lupulus* genome, has made the completion of this process now feasible in this species. We identified four novel terpene synthase enzymes in hops by interrogating the hop genome, followed by the cloning and characterisation of TPS alleles from multiple cultivars. These alleles were rapidly tested *in planta* through transient overexpression to determine their product specificity. One novel putative monoterpene synthase *HlTPS1* (unknown function) was identified. Additionally a functional monoterpene synthase *HlTPS2/HlRLS1* ((*R*)-linalool synthase 1) was discovered, as well as two novel (but related) sesquiterpene synthase genes: *HlTPS3*/*HlAFS1* and HlTPS4/*HlAFS2* (α-farnesene synthases 1 and 2). New alleles of *HlMTS1* and *HlMTS2* showed no activity *in planta,* while a new allele of *HlSTS1* (*HlSTS1-Cas-a*) produced similar products as *HlSTS1-Phx*. Analysis by qRT-PCR indicated that the expression patterns of these TPS genes are tightly developmentally controlled in cones and most genes showed strong induction of gene expression during the mid to late stages of cone development when levels of terpenes increased, but clear differences between cultivars were also observed in expression levels and patterns (Fig. 2). This will likely also be the case for key substrate pathway regulatory steps such as the DXS enzyme in the MEP pathway and the HMGR step in the mevalonate pathway, as has been reported in other plant species [49]. Results from a proteomics study in hops suggests that the MEP substrate pathway may actually be the dominant driving force for both mono- and sesquiterpene synthesis in lupulin glands [50], but this observation requires further validation.

The genomics approach applied in this study successfully identified some of the genes involved in mono- and sesquiterpene biosynthesis in hop cones. Our analysis showed that a large number of alleles were expressed in cones but a subset appears to be inactive and/or mis-spliced genes. Our analysis also showed that a moderate number of putative full-length TPS genes await further study. Characterisation of further TPS from hops will help clarify the correlation between individual TPS gene expression and the abundance of their gene products, as several mono-and sesquiterpene enzymes are competing for supply of only two substrates (GDP/FDP) derived from the MEP and/or mevalonate pathway respectively. More work also needs to be done to accurately annotate the hops genome. For example, full-length gene models for the previously cloned *HlMTS2* (β-myrcene TPS) and *HlSTS1* (α-humulene/ β-caryophyllene synthase) are absent from the genome annotation as a single gene model, but individual exons can be identified manually. A manual annotation strategy has been shown to greatly enhance the quality of automated gene models in kiwifruit [39] and could be applied to hops to improve the accuracy of gene models.

Further analysis of multiple hop genomes combined with the determination of further TPS gene functions in hops may facilitate a more informed route for genetic improvement of hops with desirable terpene profiles. For example, linalool is considered a key contributor to ‘hoppy’ flavour and there is a good correlation between fruity, floral flavour and linalool concentration [12]. Our results can be used to develop hop cultivars with novel/improved terpene profiles using marker-assisted breeding strategies to select for, or against, specific TPS alleles.

## Methods

### Plant materials

The twenty-one hop cultivars selected for the survey analysis were cultivated at the PFR Motueka Research Centre (41°5′48’’S 172°58′24’’E). A single representative pooled sample (> 50 cones) was collected from each cultivar at maturity and immediately frozen in liquid nitrogen. Cones for the cultivar developmental series using Wakatu, Wai-iti, Nelson Sauvin and ‘Nugget’ were also grown in Motueka and harvested on 17/01, 02/02, 15/02, 22/02, 02/03, 08/03 and 16/03 (Stage H0 to H6, respectively). Only H1 to H6 yielded sufficient tissue for volatile measurements, but all seven time points were included for RNA extractions. Frozen cones were ground using a cryogrinder (IKA® Model: A11 BS005) and triplicate biological samples of powder were used for RNA extraction (100 mg), for SPME (0.5 g) and solvent extraction (10 g).

### Volatile analysis by solvent extraction and SPME

Headspace volatile analysis was conducted according to previously published methods [40] with some modifications. Volatiles were collected from 0.5 g of ground hop flowers at 40°C for 12.5 min with a Gerstel Agitator controlled by an Optic 3 injection system (ATAS GL International). SPME fibres (1 cm) coated with 65 μm PDMS/DVB were used for the volatile collection. Separation was effected using a 30 m length × 0.25 mm i.d. × 0.25 μm film thickness DB-WAX (Agilent) capillary GC column in an HP6890 GC (Agilent) coupled to a Pegasus III TOF mass spectrometer (Leco). GC temperature programmes started at 50°C, 6°C∙min^−1^ to 170°C, then 20°C∙min^−1^ to 230 °C (hold 10 min). The transfer line was maintained at 240°C. Mass spectra (*m/z* 35–350) were collected at an acquisition rate of 20 spectra∙s^−1^. Terpenes in the samples were initially selected with the unique terpene single ions such as *m/z* 93, 121, 136. Other volatiles were identified by comparison with NIST V2.2, in-house mass spectral libraries and confirmed by comparison of retention indices with those of authentic standards and literature values (Table S1A). Compound peak areas were measured relative to an internal cyclohexanone standard (2.049 µg per sample).

Frozen samples for solvent extraction were defrosted into 40 mL of distilled diethyl ether, stirred for 30 min at room temperature, stored at 4 °C overnight, then frozen at − 20°C overnight. The ether extract was poured off the frozen pulp and dried over MgSO_4_, which was filtered off before the volume of the ether extract was reduced to 1 mL in a RapidVap N_2_ evaporator (Labconco, Kansas City). These samples were later diluted 100-fold for GC–MS analysis. Separations were performed on an Agilent 6890N gas chromatograph coupled to a Waters GCT time-of-flight (ToF) mass spectrometer with an EI energy of 70 eV and a scan time of 0.4 s. Splitless injections of 1 μL over 15 s were made at 220°C onto a 20 m × 0.18 mm i.d. × 0.18 μM film, DB-Wax (Agilent) capillary column with a He flow of 0.9 mL min^−1^. The oven temperature programme was 1 min at 40°C, 7°C∙min^−1^ to 240°C, which was held for 15 min. Compound concentrations were determined using diagnostic ions (for the most part *m/z* 93) against the respective synthetic external standards. Some compounds were identified by their MS fragmentation patterns and quantified against compounds with similar retention times. All external standards and hop extracts were spiked with an internal standard (pentadecafluorooctanol) (Pierce Chemicals, Rockford, IL, USA) to enable correction for extract sample volumes and changes in GC–MS system responses.

Enantiomeric analysis of linalool from solvent extracts followed previously published methods [51]. Samples were separated using a 30 m length × 0.25 mm i.d. × 0.25 µm film thickness β-Dex™ 225 (Supelco) capillary column. Injections were 1 min splitless into a helium flow of 1 ml∙min^−1^ at an injection port temperature of 220°C. The temperature programme was 1 min at 35°C, 5°C min^−1^ to 230°C, and hold for 15 min. Racemic linalool (Aldrich) and (*R*)-(-)-linalool (Fluka) were used as standards.

### Data processing and statistical analysis

Partial Least Squares-Discriminant Analysis was performed in MetaboAnalyst 5.0 (www.metaboanalyst.ca). Correlation matrices and graphical analytical analysis were generated in Excel and Origin.

### Gene identification and sequence alignments

*C. sativa* terpene synthase (TPS) gene amino acid sequences [33] were aligned to the *H. lupulus* ‘Cascade’ gene model (maskedCascadePrimary.cds.fasta, 30/05/2021- www.hopbase.com) using the TBlastn algorithm with e-value < 0.1 to identify novel hop TPS genes. Gene amino acid sequences were manually curated by comparison to the cannabis TPS protein translations (Blastp alignments) and with GenBank non-redundant protein database (Release 243) hits. Genes having the predicted protein sequence length longer than 450 AA (> 1350 in nucleotides) were classified as putative full-length TPS members. Conserved RRX8W and DDXXD motifs in the N- and C-terminus, respectively, were also discovered through this manual curation. Chloroplast targeting peptides were predicted by running the ChloroP prediction [52].

### RNA isolation, cDNA synthesis and qRT-PCR analyses

Total RNA was isolated from hop samples using the Spectrum™ Plant Total RNA kit (Sigma-Aldrich) according to the manufacturer’s instructions. RNA concentration and quality were determined with a Nanodrop spectrophotometer (Thermo Fisher Scientific) and by gel electrophoresis. First-strand cDNA was synthesised from 1 µg total RNA using a QuantiTect Reverse Transcription Kit (QIAGEN) and diluted 50-fold before use. RT-qPCR of hop TPS genes and the reference gene *DRH1* [37, 38] were performed on a LightCycler 480 platform using SsoAdvanced™ Universal SYBR® Green Supermix (BioRad). Results were analysed using the LightCycler 480 software (Roche Applied Science). Program: 5 min at 96°C; 40 cycles of 10 s at 95°C, 10 s at 60°C, and 20 s at 72°C; followed by melting curve analysis: 95°C 5 s, 65°C 60 s, then ramping at 0.18°C∙s^−1^ to 95°C [53]. Primers are listed in Table S5.

### Transient expression of terpene synthases in *N. benthamiana*

Putative TPS genes were Gateway BP cloned into pDONR221 (Invitrogen) using the primers described in Table S5 and then transferred by Gateway LR reactions into the binary destination vector pHEX2 according to manufacturer’s instructions (Invitrogen). pHEX2 contains the *Cauliflower Mosaic Virus (CaMV)* 35S promoter and octopine synthase terminator [36]. Over-expression constructs were electroporated into *A. tumefaciens* strain GV3101. Infiltrations into *N. benthamiana* leaves were carried out as described previously [51] and all TPS genes were co-infiltrated with the P19 silencing suppressor [53]. A pHEX2-GUS construct was used as a negative control. Samples were analysed at 7 days post infiltration. Volatiles were collected using SPME [54] from 2 g of tissue or extracted into the solvent pentane/Et_2_O at 2 mL∙g^−1^ FW. GC–MS analysis was performed as described previously [51]. The use of the AcDXS and HMGR construct have been previously described [55, 56].

## Supplementary Information

**Additional file 1:** **Figure S1A.** Amino acid alignment of published and novel terpene synthases from hops. **Figure S1B.** Phylogenetic tree of published and novel terpene synthases from hops.

**Additional file 2:** **Table S1A.** Compounds detected in ripe cones of twenty-one hop cultivars. **Table S1B.** Correlation matrix of volatile compounds produced in cones of 21 hops cultivars.  **Table S2.** Published and cloned Humulus lupulus terpene synthase genes. **Table S3A.** Hops gene models predicted coding sequence, GenBank hit parameters and conserved motifs for putative mono-, sesqui- and diterpene synthases. **Table S4.** Transient *Agrobacterium* expression of HlTPS genes in *Nicotiana benthamiana*. **Table S5.** Primers used for Gateway cloning and qPCR.

## Data Availability

The datasets supporting the conclusions of this article are included within the article and its additional files. GenBank sequences reported in this article can be found under accession numbers OQ096016-OQ096028 and are listed in Table S2C.
