# Supplementary material for: The hops (Humulus lupulus) genome contains a mid-sized terpene synthase family that shows wide functional and allelic diversity
Source: BMC Plant Biol. 2023 May 26;23:280. doi: 10.1186/s12870-023-04283-y (PMC10214682; doi:10.1186/s12870-023-04283-y)
Supplement: Supplementary file 1 — Additional file 1: Figure S1A. Amino acid alignment of published and novel terpene synthases from hops. Figure S1B. Phylogenetic tree of published and novel terpene synthases from hops. [file 12870_2023_4283_MOESM1_ESM.docx]

**Figure S1A:** Amino acid alignment of published and novel terpene synthases from hops.


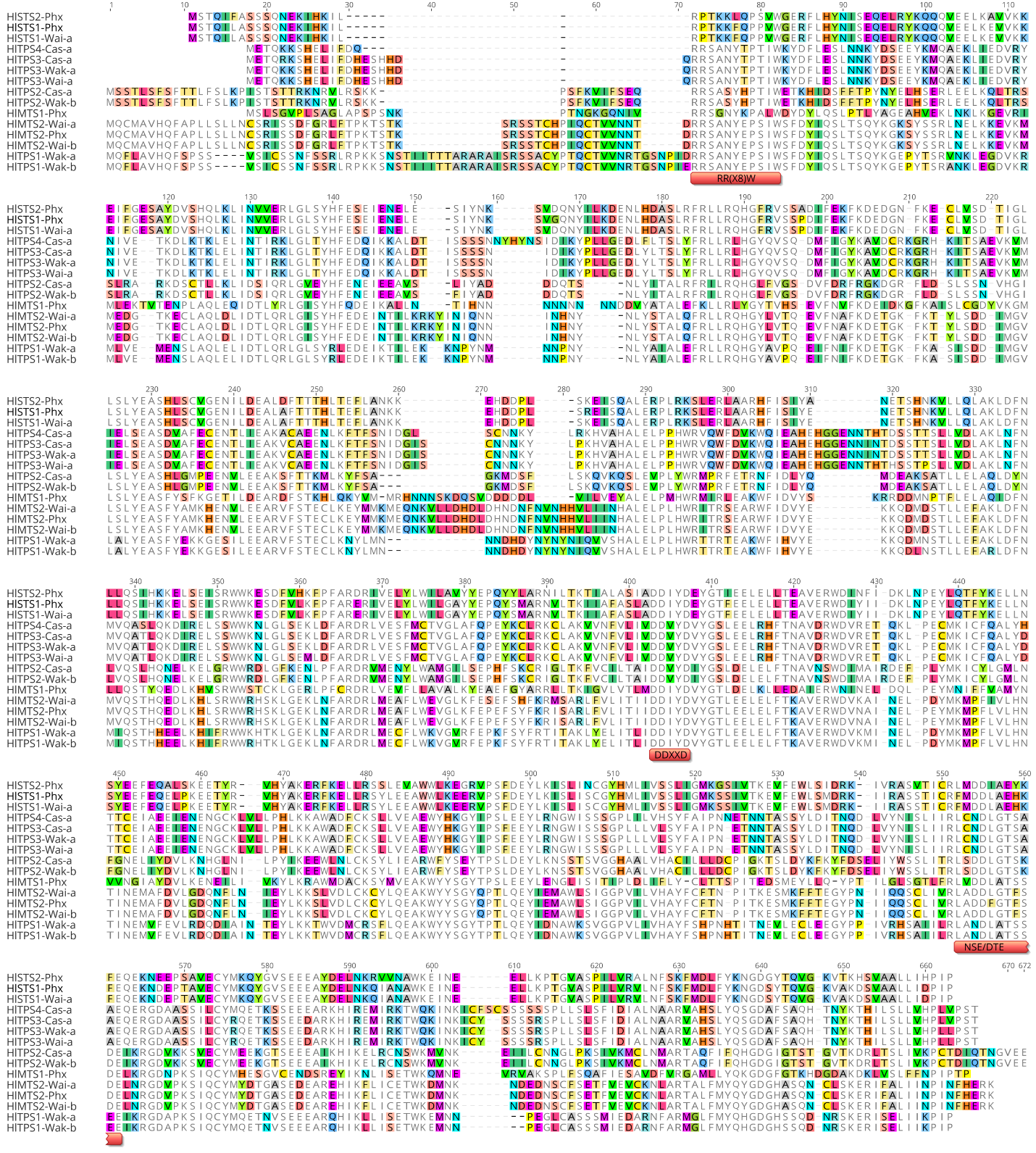


**(A)** Proteins were aligned using the Muscle algorithm in Geneious using default parameterswww.geneious.com. The conserved RR(X8)W N-terminal domain and DDXXD signature catalytic domain are underlined in red. The NSE/DTE metal binding motif (Christianson, 2006; Zhou and Peters, 2009) is based the consensus: (L,V)(V,L,A)(N,D)D(L,I,V)x(S,T,G)xxxE and underlined in red.

Published genes from ‘Phoenix’ HlMTS1-Phx; HlMTS2-Phx; HlSTS1-Phx and HlSTS2-Phx (GenBank: EU760348-EU760351). Novel HlTPS1–4 alleles from: Wakatu™ (Wak); Wai-iti™ (Wai), and ‘Cascade’/Taiheke® (Cas). Disagreements to the consensus are highlighted in colour.

**Figure S1B:** Phylogenetic tree of published and novel terpene synthases from hops.


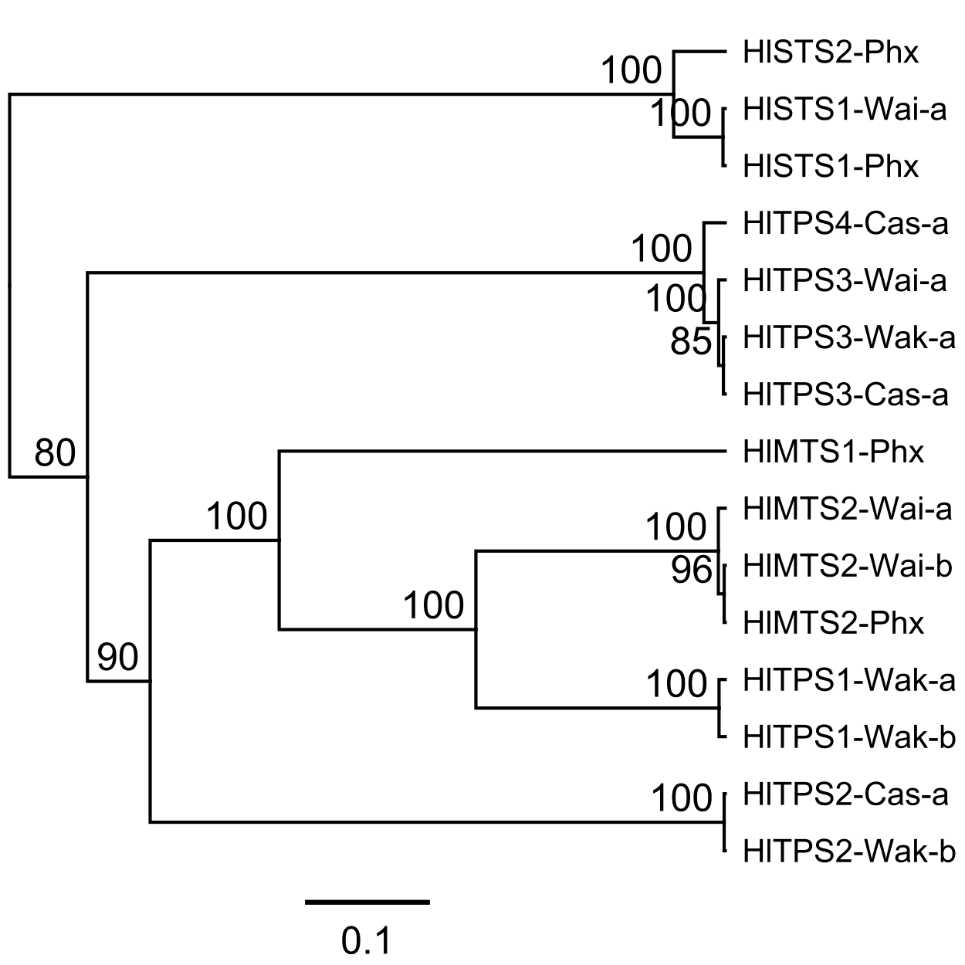


**(B)** The tree was generated using Geneious Treebuilder with the UPGMA tree builder tool and Jukes-Cantor distance matrix using default parameters and 1000 bootstrap replicates with a support threshold of >70%. The scale bar/pairwise distance is defined as the fraction of mismatches across the aligned positions.

**Christianson DW.** 2006. Structural biology and chemistry of the terpenoid cyclases. *Chemical Reviews* **106**, 3412-3442 doi: 10.1021/cr050286w.

**Zhou K, Peters RJ.** 2009. Investigating the conservation pattern of a putative second terpene synthase divalent metal binding motif in plants. *Phytochemistry* **70**, 366-369 doi: 10.1016/j.phytochem.2008.12.022.
